# Fistulating diverticulitis: a distinct clinical entity?

**DOI:** 10.3389/fmed.2025.1500053

**Published:** 2025-03-13

**Authors:** Savvas Papagrigoriadis, Giovanni Brandimarte, Antonio Tursi

**Affiliations:** ^1^IASO Hospital, Athens, Greece; ^2^One Welbeck Digestive Health Centre, London, United Kingdom; ^3^Division of Internal Medicine and Gastroenterology, “Cristo Re” Hospital, Rome, Italy; ^4^Territorial Gastroenterology Service, Barletta-Andria-Trani Local Health Agency, Andria, Italy; ^5^Department of Medical and Surgical Sciences, School of Medicine, Catholic University, Rome, Italy

**Keywords:** diverticulitis, fistula, diverticular disease, surgery, colovesical fistula, colovaginal fistula, enterocutaneous fistula, colofallopian fistula

## Abstract

**Introduction:**

Diverticular disease (DD) of the colon has a number of phenotypes, including asymptomatic diverticulosis and complicated diverticulitis with bowel perforation or bleeding. The factor that affects the phenotype of this condition and leads to a wide range of clinical presentations is unknown. The formation of fistulas associated with diverticulitis has long been recognized, and they are treated according to *ad hoc* indications. We hypothesized that the formation of fistulas in diverticular disease exhibits such a wide range of variable anatomic features that it may be considered a distinct form of the condition, fistulating diverticulitis (FD).

**Methods:**

We conducted a narrative review based on 50 years of publications covering a wide range of diverticulitis-associated fistulas, both common and uncommon.

**Results:**

While there is abundant literature on common fistulas, such as colovesical and colovaginal fistulas, little is known about rarer fistulas, such as coloenteric fistulas, colocutaneous fistulas, and genitourinary tract fistulas. The majority of these fistulas are treated surgically, which is in contrast to the trend toward conservative management that is predominant in acute or chronic diverticulitis.

**Discussion:**

Epidemiological and histological evidence support the hypothesis that FD may be a feature of chronic DD that requires individual management. Histopathology shows similarities with Crohn’s disease. It remains unknown which underlying immune or genetic factors may be affecting the clinical presentation of these patients, leading to fistulation. We contend that there is adequate published evidence to characterize a distinct phenotype of FD that can involve the entire GI tract and other organs. Surgical guidelines may need to be modified to treat this small but important group, which predominantly requires surgical treatment.

## Introduction

Fistulas are defined as abnormal communication between two epithelial surfaces. These epithelial surfaces are often found in an organ or on the skin (which is technically also considered an organ) (1).

Several bowel conditions cause fistulation complications at every site of the gastrointestinal tract. The underlying cause is inflammation, which can occur with or without sepsis. Typical fistulating bowel conditions include Crohn’s disease and Actinomycosis, although fistulas can also form due to factors such as radiotherapy, cancer, and abdominal abscesses.

Diverticular disease (DD) is one of the most common diseases worldwide (2), and it is often a leading cause of fistulas, such as colovesical, colovaginal, coloenteric, colocutaneous, colouterine, and several uncommon types.

Fistulas in DD may occur because, like Crohn’s disease, it is a transmural disease (3) in which the extension of the inflammation outside the colonic wall can lead to abnormal inflammatory communication with other organs.

The frequency and range of fistula formation in DD are so extensive and distinct in terms of clinical presentation and its consequences that they justify consideration as a separate phenotype of the condition: fistulating diverticulitis (FD). It is a well-known clinical entity among clinicians with experience in diverticulitis, but clinical manifestations and severity vary greatly depending on the anatomic sites involved and the degree of inflammation or sepsis. Fistulas in DD are uncommon compared to other clinical manifestations of the condition. A large epidemiological study in the United States, involving more than 7 million admissions, found that diverticular-related fistulas were responsible for more than 3% of DD admissions (4). Given that admission for DD is a common hospital event, the absolute number of FD cases is such that any clinician in gastroenterology or surgery is likely to encounter them relatively frequently. The same study found that surgery was the most common treatment choice for FD in 64% of cases. This frequency of surgical indication is supported by Psarras et al., estimating that fistulation accounts for 5–9% of DD cases that require surgery (5).

FD cannot be managed within the standard protocols for treating left-sided diverticulitis, as treatment is less often conservative and requires diagnostic investigations tailored to the anatomic features of the presentation. For example, a fistula in the abdominal wall requires CT scan imaging, a colovesical fistula necessitates cystoscopy, and a diverticular fistula extending into the perineum is best evaluated with magnetic resonance imaging (MRI). Clearly, an accurate differential diagnosis of other pathologies that can cause fistulas, such as Crohn’s disease and malignancy, is essential.

Some believe the FD phenotype has a distinct “virulence” characterized by severe symptoms, a high rate of complications, frequent recurrences, and an increased risk of reoperation. This has caused some authors to refer to it as *“malignant diverticulitis*,” which does not imply neoplasia but rather a non-benign course with a higher recurrence and greater risk than typical cases (6, 7).

## Methods

We conducted a literature review on PubMed, Scopus, and Embase for clinical presentations of fistulas associated with DD from 1974 to 2024. Our review aimed to assess the range of clinical presentations, both common and uncommon, to draw the outline of a distinct phenotype of diverticulitis. As this was a heterogeneous group of interest, we did not attempt to compare clinical outcomes; therefore, a narrative review was preferred over a systematic review. A selection of representative articles was made by three authors who are specialists in DD and was compiled into the narrative review.

## Results

Fistulas in DD are not only uncommon—some are more common, while others are uncommon (Table 1).

**Table 1:** Type of fistulas occurring in diverticular disease.

Common	Uncommon	Very uncommon
Colovesical	Coloenteric	Sigmoidocutaneous
Colovaginal	Colocutaneous	Renocolic-cutaneous
		Necrotizing fasciitis
		Groing hernia
		Infection of the scrotum
		Aortosigmoid
		Thrombophlebitis of the inferior mesenteric vein
		Colosplenic
		Colpo-salpingeal
		Tuboovarian abscess
		Femoral osteomyelitis
		Hepato-bronchial

### Common fistulas

#### Colovesical fistulas

By far, the most common type of fistula caused by diverticulitis is colovesical fistula. The incidence of colovesical fistulas is estimated to be 2–4% (8). It is not uncommon for colovesical fistulas to be the first manifestation of diverticulitis. The typical presentation of a colovesical fistula includes urosepsis, pneumaturia, and fecaluria. Diagnosis is confirmed through imaging techniques such as CT scan, cystography, cystoscopy, and sigmoidoscopy. However, the most accurate method for confirming the fistula is *the poppy seed test*, which involves eating a handful of poppy seeds mixed with yoghurt and observing their passage in the urine within a few hours. Melchior et al. examined the diagnostic sensitivity for a colovesical fistula and found the following: poppy seed test 94.6%, CT scan 61%, MRI scan 60%, cystogram 16.7%, contrast enema 35.7%, cystoscopy 10.2%, and colonoscopy 8.2% (9).

Laparotomy or laparoscopy can be used for the treatment of colovesical fistulas, with the choice depending on the experience of the surgeons. Several series of laparoscopic operations for colovesical fistulas have now been published in the literature, demonstrating the feasibility and safety of the technique (10–12). The published results of laparoscopy have been generally excellent, with very low stoma rates (13). A systematic review of 11 non-randomized studies on laparoscopic surgery for colovesical fistulas found very low recurrence (0.8%) and reintervention rates (2%) and concluded that, despite the absence of randomized trials, laparoscopic surgery appeared to be safe (14). Regardless of the surgical approach chosen, it is important to prioritize the preservation of the ureters. No patient should go to the operating theater without a CT scan, which, among other things, will also show if there is any hydronephrosis. If even minor hydronephrosis is present, then cystoscopy and pigtail stenting must be performed at the start of the operation (15). However, most surgeons would take the precaution of placing the stents in any case, and studies have confirmed that there is a trend in this direction (16).

If the ureters are involved in the inflammatory mass, a urologist should be involved in the operation to free the ureters and ensure proper bladder repair following the excision of the fistula. However, in most cases, only the dome of the bladder is affected, and a general/colorectal surgeon can “scrape off” the sigmoid colon from the bladder, addressing a small bladder dome defect that can be repaired with two layers of sutures. Minimal dissection and mobilization of the bladder are required, provided that there is certainty about the safety of the ureteric orifices (17). The bladder is filled and checked for leaks at the end of the operation, and the urinary catheter remains in place for a week. A cystogram is performed prior to removing the catheter at the end of the week. Some believe that bladder repair is unnecessary as fistula heals quickly after the excision of the fistula. However, repairing the dome in two layers is easy, so there is no reason not to perform the repair.

#### Colovaginal fistula

A colovaginal fistula occurs only if there has been a prior hysterectomy because it is only then that the sigmoid colon can lie on the vaginal stump and erode, forming a fistula (18). Although they present a lower risk of severe sepsis than colovesical fistulas, they can cause symptoms that are devastating to quality of life. Fecal and mucous discharge, as well as bleeding and mucous, occur per vagina. In addition to uncontrollable fecal incontinence through the vagina, there are also erosions, irritation, and pain from the vagina that is difficult to manage. Sexual intercourse is impossible. A pelvic abscess can, in some cases, form and expand. Surgical repair is the only treatment option, though transvaginal closure has been reported as an option (19).

In both colovesical and colovaginal fistulas, the most common treatment is anterior resection of the sigmoid and upper rectum with primary anastomosis. When the anastomosis is below the promontory, and there is active inflammation or pus, a defunctioning stoma may be recommended. Laparoscopic surgery has been demonstrated to be feasible and curative for these fistulas, with a reasonable conversion rate, when performed by experienced laparoscopists. Garcea et al. (20) reported their experience with 90 consecutive cases of colovesical fistulas. Pneumaturia or fecaluria was the mode of presentation in 90% of cases. A total of 72 of these fistulas were caused by diverticular disease, with the rest resulting from malignancy or Crohn’s disease. Primary anastomosis was achieved in 92% of cases. Bartus et al. reported a series of 40 consecutive diverticular fistulas, 36 of which were treated laparoscopically. The conversion rate was 25%, however the remaining procedures were performed laparoscopically without complications (10). This study showed that, despite the high conversion rate, the majority of patients with fistulas can benefit from laparoscopic surgery.

### Uncommon fistulas

#### Coloenteric fistulas

Coloenteric fistulas are formed when a ruptured diverticulum causes an abscess that erodes into an adjacent small bowel loop (21, 22). Similarly, a diverticular fistula between the sigmoid and the caecum has been described (23). Small bowel obstruction has been described as a result of the adherence of loops to the inflamed sigmoid colon (24).

#### *Colocutaneous* fistulas

These fistulas are more commonly found in the abdominal wall or thigh (25–28). Although a fistula is most commonly found in the left thigh, it has also been observed in the right thigh (29). Thigh fistulas can sometimes be misjudged for superficial cutaneous “boils” and left untreated until they cause necrotizing fasciitis of the limbs (30, 31). Laparoscopic insufflation can be used to confirm the existence of a colocutaneous fistula (32). The sigmoid colon is not the only origin of a colocutaneous fistula, as it has also been described in other parts of the colon, such as the transverse colon (33).

A colonic fistula is known to be a risk after CT scan-guided drainage. Raman et al. published a series involving 105 patients who underwent CT scan drainage of diverticular abscesses, and 55% of them subsequently developed fistulous communication between the colon and the drain placed by the radiologist (34). This serves as a warning about the dangers of percutaneous drains.

#### Other very uncommon fistulas

***Sigmoidocutaneous*** fistulas presenting as perianal abscesses are another unusual presentation (35).

Inflammation from kidney disease, combined with diverticulitis, has been reported to **cutaneous renocolic** fistulas (36).

***Necrotizing fasciitis*** has been reported as the initial presentation of diverticulitis that fistulises to the abdominal wall (32, 37).

A diverticular fistula or abscess presenting ***as a groin hernia*** has been described (38, 39), which may even resemble acute incarceration (40, 41).

Rarely fistulas can track into the scrotum via the inguinal canal and present as an acute ***infection of the scrotum*** (42). There has even been a description of emphysematous epididymitis as a result of sigmoid diverticulitis (43). The fistula track may instead proceed along the ischiorectal fossa and present as a perianal abscess (44) or a supra-levator abscess (45).

A very uncommon complication of sigmoid diverticulitis is the development of a fistula in major arteries, such as the aorta. An ***aortosigmoid fistula*** results in catastrophic bleeding with very high mortality. A CT scan is used to diagnose the condition, and treatment involves emergency colectomy and salvage-covered stent placement as interval treatment (46).

A presentation with portomesenteric gas and sepsis has been described in cases of fistulisation of the inferior mesenteric vein due to diverticulitis (47, 48).

Septic ***thrombophlebitis of the inferior mesenteric vein***, which does not respond to antibiotics, is a uncommon complication of diverticulitis. It is diagnosed using a CT scan with IV contrast (49), which reveals gas in the inferior mesenteric vein (50–52). If the condition has a poor response to antibiotics, emergency colectomy is required.

A diverticular fistula to the spleen, known as a ***colosplenic*** fistula, is very uncommon, with only a few cases reported (53).

In the presence of a meningocele that communicates with a diverticular abscess of the sigmoid colon, meningitis can result (54).

All diverticular fistulas to the skin should be treated following the same principles as other bowel fistulas. As these fistulas have low output, many will heal with conservative treatment, including vacuum-assisted closure (VAC) therapy and endoscopic organic glue application. The remaining ones typically have a good outcome with surgery (55, 56).

Unexpected air in the uterus along with diverticulitis may reveal a uncommon ***colpo-salpingeal fistula,*** which is treated with laparotomy, colectomy, salpingectomy, and potentially hysterectomy (57–59). Diverticulitis can also present as a tubo-ovarian abscess (60).

Fistulas communication with the left hip joint has given rise to ***femoral osteomyelitis*** secondary to sigmoid diverticulitis (61).

Finally, a case of ***hepato-bronchial fistulisation*** resulting from sigmoid diverticulitis has been described (62).

## Discussion

DD is a common cause of fistulas, with a wide range of severity. The more frequent ones, such as colovesical or colovaginal fistulas, are easy to suspect and diagnose. However, clinicians should maintain a high level of clinical suspicion for unusual presentations of abdominal fistulas.

Evidence on the pathology of DD suggests that it is a transmural condition that involves a number of individual features that promote the development of fistulas. Burroughs et al. reported granulomatous inflammation and granulomatous vasculitis with mural lymphoid aggregates, which resemble but are distinct from Crohn’s disease (63). Goldstein et al. showed non-necrotizing granulomatous inflammation situated at the outer edge of the muscularis propria. Another feature was active vasculitis with dense lymphoplasmacytic inflammation transmurally (64).

These are features of chronic inflammation that may signify a different pathogenesis process than an acute peri-diverticulitis abscess, which is believed to occur due to the obstruction of a diverticulum by faecoliths and others. Several authors have commented on the remarkable histological similarity between chronic diverticulitis and Crohn’s disease, from which it is distinguished by rectal sparing, a universal feature of DD (65–67). This reinforces the hypothesis of the role of chronic inflammation in DD, as postulated by some authors (68, 69).

A review of the literature associated fistulation with the chronicity of DD and may influence the advice on monitoring and managing chronic DD for prevention. Until now, however, acute diverticulitis that has guided the design of management strategies.

FD has not been reported to respond well to conservative treatment, and for this reason, the current surgical guidelines, which advise only individualized decisions on the suitability for surgery, might need to be modified to identify this important small group as likely to benefit from surgery. Fortunately, surgical treatment seems to yield good results in FD, and this is true also for minimally invasive laparoscopic and robotic surgery (70).

Kitaguchi et al. published a series of 11 cases of laparoscopic surgery for a diverticular colovesical fistula (71). The conversion rate to open surgery was 27%, with postoperative minor complications occurring in 36% of cases, but no major complications.

Rizzuto et al. (72) published a retrospective case–control study comparing laparoscopic and open resection for colovesical fistulas in diverticular disease, involving 76 patients. They found that laparoscopy had advantages in terms of blood loss, postoperative ileus, and length of stay. There were no fistula recurrences at 2 years.

Haass et al. (73) published a study of 190 robotic colonic resections for diverticulitis that were performed as hybrid “NICE” procedures. This approach combines robotic surgery with transanal specimen extraction (NOTES). Of these procedures, 47.8% involved complicated diseases, including fistulas, abscesses, or strictures. The fistula-involving resections were similar to the uncomplicated ones in terms of intracorporeal anastomosis formation, postoperative complications, and length of hospital stay.

Abbas et al. published a series involving 42 patients who underwent laparoscopic resection for diverticular fistulas, including colovesical, colovaginal, and colocutaneous (74). A chronic abscess was present in one-third of the cases. There was a low conversion rate of 10%, and the rate of diverting stoma was 5%. There were no fatalities. However, one ureteric injury was reported.

It therefore appears that there are no technical contraindications to using the latest minimally invasive surgical techniques in FD, provided that the appropriate surgical expertise is available.

## Conclusion

We conclude that FD has enough distinguishing features to be considered a distinct form of DD, which is characterized by (a) a background of chronic inflammation, (b) a higher rate of complications, and (c) a more frequent need for surgical treatment. Surgical guidelines may need to be modified to recommend surgery as the predominant treatment for this small but clinically important group.
